# *Staphylococcus aureus* foldase PrsA contributes to the folding and secretion of protein A

**DOI:** 10.1186/s12866-024-03268-7

**Published:** 2024-04-02

**Authors:** Mei-Hui Lin, Chao-Chin Liu, Chiao-Wen Lu, Jwu-Ching Shu

**Affiliations:** 1 Graduate Institute of Medical Biotechnology and Laboratory Science, College of Medicine, Chang Gung University, Tao-Yuan, 333 Taiwan; 2 Department of Laboratory Medicine, Chang Gung Memorial Hospital at Linkou, Tao-Yuan, 333 Taiwan; 3 Graduate Institute of Biomedical Sciences, College of Medicine, Chang Gung University, Tao- Yuan, 333 Taiwan

**Keywords:** *Staphylococcus aureus*, Foldase, PrsA, Protein A

## Abstract

**Background:**

*Staphylococcus aureus* secretes a variety of proteins including virulence factors that cause diseases. PrsA, encoded by many Gram-positive bacteria, is a membrane-anchored lipoprotein that functions as a foldase to assist in post-translocational folding and helps maintain the stability of secreted proteins. Our earlier proteomic studies found that PrsA is required for the secretion of protein A, an immunoglobulin-binding protein that contributes to host immune evasion. This study aims to investigate how PrsA influences protein A secretion.

**Results:**

We found that in comparison with the parental strain HG001, the *prsA*-deletion mutant HG001Δ*prsA* secreted less protein A. Deleting *prsA* also decreased the stability of exported protein A. Pulldown assays indicated that PrsA interacts with protein A in vivo. The domains in PrsA that interact with protein A are mapped to both the N- and C-terminal regions (NC domains). Additionally, the NC domains are essential for promoting PrsA dimerization. Furthermore, an immunoglobulin-binding assay revealed that, compared to the parental strain HG001, fewer immunoglobulins bound to the surface of the mutant strain HG001Δ*prsA*.

**Conclusions:**

This study demonstrates that PrsA is critical for the folding and secretion of protein A. The information derived from this study provides a better understanding of virulent protein export pathways that are crucial to the pathogenicity of *S. aureus*.

**Supplementary Information:**

The online version contains supplementary material available at 10.1186/s12866-024-03268-7.

## Introduction

Gram-positive bacteria have only a single membrane that is surrounded by a thick cell wall. Therefore, the secreted proteins are first transported across the cytoplasmic membrane into the space between the cell membrane and the cell wall. As is generally known, the majority of proteins translocated across the membrane remain in an unfolded state [1]. After translocation, the proteins are folded into their correct conformation to protect them from degradation by “quality control” proteases at the cytoplasmic membrane-cell wall interface [2].

PrsA, a parvulin-type peptidyl-prolyl *cis/trans*-isomerase (PPIase) family member, is found in many Gram-positive bacteria and functions as a chaperone or foldase [3]. Theoretically, PrsA does not influence the expression or transport of exoproteins but is required for their proper folding and stability [4, 5]. In *Bacillus subtilis*, PrsA is essential for bacterial viability and the late stage of protein secretion [6]. It has been demonstrated that α-amylase, β-glucanase and β-lactamase are exported in a PrsA-dependent manner [4]. PrsA also folds and stabilizes penicillin-binding protein 2a (PBP2a) of *B. subtilis* [7]. In addition, PrsA has been shown to impact the virulence of diverse microorganisms. The secretion of protective antigen (PA), a component of the *Bacillus anthracis* edema and lethal toxins, is dependent on PrsA [8]. Two PrsA (PrsA1 and PrsA2) proteins are present in group A *Streptococcus* (GAS), and deletion of PrsA reduces SpeB maturation and decreases the virulence of GAS in vivo [9, 10]. *Listeria monocytogenes* also contains PrsA1 and PrsA2 [11, 12]. PrsA2 is required for the secretion of several virulence factors, such as listeriolysin O (LLO), metalloprotease (Mpl), and phospholipase [11, 13, 14]. In *Streptococcus mutans*, PrsA plays crucial roles in modulating cell surface characteristics and is involved in the secretion of AtlA and biofilm formation [15, 16].

*Staphylococcus aureus* is an important pathogen that causes a variety of human infections. These infections are attributed to the production and secretion of numerous virulence factors by this organism [17]. Similar to other Gram-positive bacteria, *S. aureus* expresses PrsA, but it is not essential for *S. aureus* viability [18, 19]. The expression of PrsA is upregulated upon cell wall stress by the VraRS two-component system and is involved in both glycopeptide and oxacillin resistance [18, 20]. In addition to acting as a molecular chaperone, PrsA plays a role in membrane lipid remodeling and is involved in daptomycin-mediated β-lactam resensitization [21]. A recent study indicated that genomic variation within *prsA* is associated with *S. aureus* adaptation to healthcare environments [22]. Our earlier study found that deletion of *prsA* in *S. aureus* HG001 altered the exoproteome and reduced the secretion of protein A, a virulence factor involved in host immune evasion [23]. In this study, we investigated the mechanism by which PrsA affects the secretion of protein A. The results demonstrated that PrsA formed a dimer and interacted with protein A. Deficiency of PrsA decreased the stability of protein A and reduced the amount of secreted protein A, leading to a decrease in the binding of immunoglobulins to *S. aureus*. The results suggest that PrsA is critical for the secretion of protein A and is involved in regulating the virulence of *S. aureus*.

## Results

### PrsA and protein A secretion

**Fig. 1:**
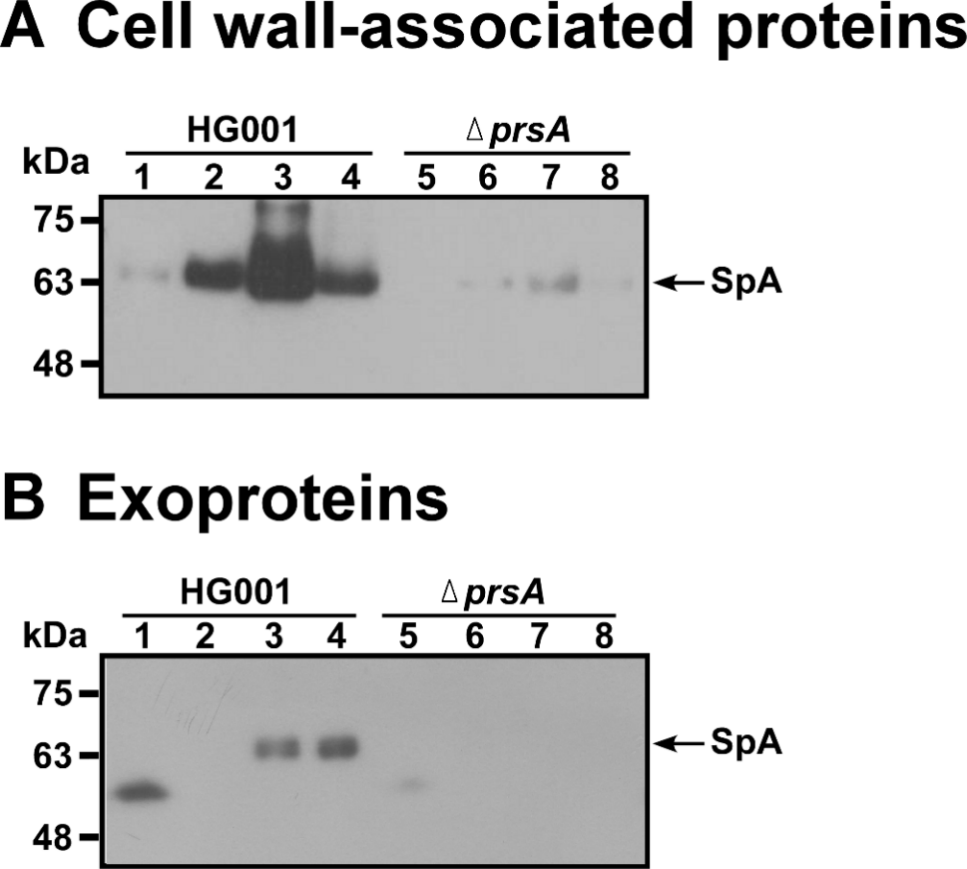
Secretion of SpA by *S. aureus*. *S. aureus* HG001 and *S. aureus* HG001Δ*prsA* were cultured in TSB for 1 h (lanes 1 and 5), 3 h (lanes 2 and 6), 5 h (lanes 3 and 7), and 7 h (lanes 4 and 8). (**A**) Cell wall-associated proteins were prepared from 5 × 10^8^ CFU of cells, and SpA in the fraction was analyzed by immunoblotting using anti-SpA antibody. (**B**) After culturing the cells, the proteins in the culture medium were concentrated using Amicon Ultra centrifugal filters and detected by immunoblotting. The cell lysates prepared from 5 × 10^8^ CFU of cells were separated by SDS-PAGE and electrotransferred onto PVDF membranes. The PVDF membranes were stained with 0.2% Ponceau S solution as a loading control to ensure the equivalent protein content extracted from an equal number of cells (Supplemental Fig. S1)

Staphylococcal protein A (SpA) is primarily anchored to the cell wall of *S. aureus*, although some of it is released into the culture medium [24]. In an earlier proteomic study, we found that although SpA was present in the cell wall fractions and culture medium of *S. aureus* HG001, the abundance of SpA in the cell wall and the culture medium decreased significantly after *prsA* was deleted, showing that PrsA critically influences the secretion of SpA [19]. In this study, we prepared the proteins from cell wall fractions of *S. aureus* HG001 and HG001Δ*prsA* that had been cultured for 1 h, 3 h, 5 h and 7 h, and conducted an immunoblotting study to elucidate the impact of *prsA* deletion on SpA secretion under different culture conditions. The results showed that in *S. aureus* HG001, only a small amount of SpA was detected at 1 h after inoculation (Fig. 1A, lane 1), and the amount increased significantly at hour 3, reached a maximum level at hour 5 (Fig. 1A, lanes 2, 3), and then decreased at hour 7 (Fig. 1A, lane 4). The abundant expression of SpA at hour 5, i.e., the exponential phase of growth, is consistent with the fact that translation of SpA is inhibited after this stage of growth [25]. Meanwhile, SpA was barely detectable in the cell wall of *S. aureus* HG001Δ*prsA* (Fig. 1A, lanes 5–8). Our study also showed that SpA was secreted by *S. aureus* HG001 and accumulated in the culture medium; the protein was detectable by immunoblotting at 5 and 7 h after inoculation (Fig. 1B, lanes 3, 4). On the other hand, little SpA was secreted by *S. aureus* HG001Δ*prsA* (Fig. 1B, lanes 5–8). Notably, a protein with a molecular weight smaller than SpA was nonspecifically detected by an anti-SpA antibody at 1 h after inoculation. The identity of this protein is unknown.

### *PrsA* deletion destabilizes protein A

**Fig. 2:**
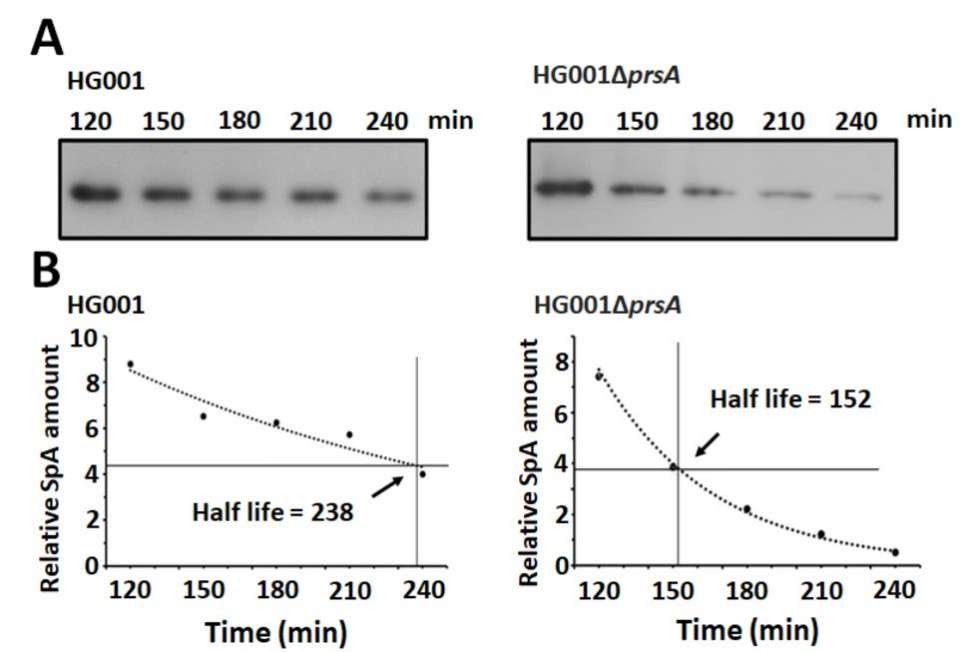
Stability of SpA exported to the cell wall. (**A**) *S. aureus* HG001 and HG001Δ*prsA* were cultured for 5 h and treated with erythromycin to inhibit protein synthesis. The cell wall-associated proteins were then extracted at the time indicated after adding erythromycin. The amount of SpA was determined by immunoblotting. (**B**) Bands from the experiments in panel A were quantified using a densitometer. The half-life of SpA was calculated from the analysis of the exponential regression curve using the GraphPad Prism software. The cell lysates prepared from 5 × 10^8^ CFU of cells were separated by SDS-PAGE and electrotransferred onto PVDF membranes. The PVDF membranes were stained with 0.2% Ponceau S solution as a loading control to ensure the equivalent protein content extracted from an equal number of cells (Supplemental Fig. S2)

Unfolded secretory proteins are known to be susceptible to proteolytic degradation [2, 26]. If PrsA is involved in the folding of SpA, in the absence of PrsA, unfolded SpA is likely degraded by protease. Therefore, in this study, we determined the stability of SpA in *S. aureus* HG001 and HG001Δ*prsA*. The cells were cultured for 5 h, when the largest amount of SpA was secreted (Fig. 1A), and then treated with erythromycin to inhibit global protein synthesis. After erythromycin treatment, the amount of cell wall-associated SpA was then monitored for 4 h. The band intensity from western blot films was analyzed. Due to a significantly lower expression of SpA in HG001Δ*prsA* compared to the wild-type strain HG001, we examined the proteins extracted from HG001Δ*prsA* with a sample volume fivefold larger than that of proteins extracted from the wild-type strain HG001. This approach aimed to better elucidate the trend of SpA degradation in HG001Δ*prsA*. Moreover, given the sustained stability of SpA over several hours [27], there was no change in the intensity of the SpA bands on the gel during the initial 2 h of this experiment. Therefore, to obtain a more reliable regression curve for calculating the relative half-life, SpA degradation was monitored starting from 2 h after adding erythromycin. The results showed that SpA was less stable in a genetic background without *prsA* (Fig. 2A). The half-life of SpA from *S. aureus* HG001 was 238 min, while in *S. aureus* HG001Δ*prsA*, it was 152 min. The results validate our hypothesis and demonstrate the significant impact of *prsA* deletion on the stability of secreted SpA.

### Interaction between SpA and PrsA in vivo

**Fig. 3:**
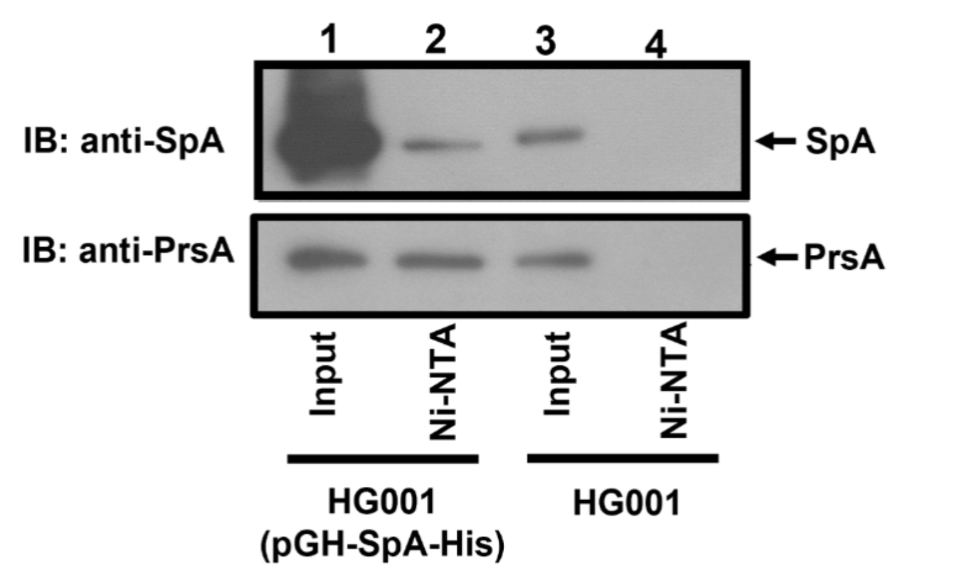
Interaction between PrsA and SpA. Ni-NTA beads were added to a protein mixture extracted from the cell wall of *S. aureus* HG001(pGH-SpA-His) (lanes 2) and *S. aureus* HG001 (lanes 4). Proteins that were pulled down by the beads were analyzed by immunoblotting using anti-SpA (upper panel) and anti-PrsA antibodies (lower panel). Lanes 1 and 3 were loaded with 10% cell wall extracts. The full-length blots were shown in Supplemental Fig. S2

PrsA is known to act as a chaperone in *Bacillus subtilis* [28]; if PrsA in *S. aureus* has a similar function, it may interact with SpA to assist in its post-translocational folding. To demonstrate the interaction, we transformed *S. aureus* HG001 with pGH-SpA-His, which expresses a histidine-tagged SpA (His-SpA). Since the interaction between SpA and PrsA likely occurs in the region between the cell membrane and cell wall, we purified cell wall-associated proteins from *S. aureus* HG001(pGH-SpA-His). Ni-NTA agarose beads were used to capture proteins that interact with His-SpA fusion proteins. The results showed that PrsA in the protein mixture isolated from *S. aureus* HG001(pGH-SpA-His) (Fig. 3, lanes 1) was retained by His-SpA-Ni-NTA agarose beads (Fig. 3, lanes 2). In a negative control, SpA and PrsA in the protein mixture isolated from *S. aureus* HG001 did not bind to the beads (Fig. 3, lanes 4). The pulldown of PrsA with His-SpA-Ni-NTA agarose beads indicated that PrsA interacts with SpA in vivo.

### Determining the domains in PrsA required for interaction with SpA

**Fig. 4:**
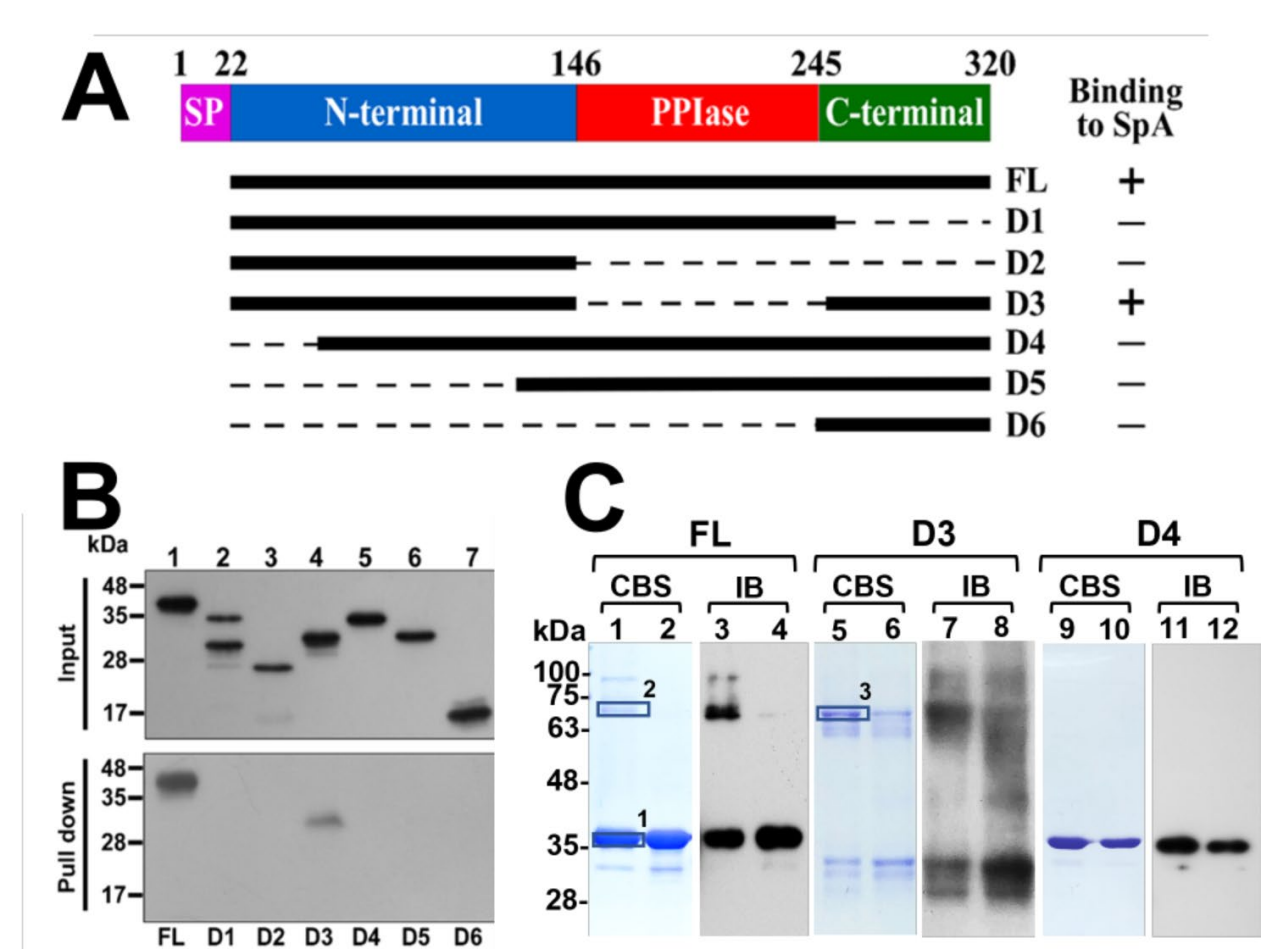
Mapping the regions in PrsA that interact with SpA and the formation of the PrsA dimer. (**A**) Map of PrsA and its truncated mutants. Based on the sequence homology with the *B. subtilis* PrsA protein, PrsA from *S. aureus* HG001 contains a signal peptide (SP), an N-terminal, a PPIase, and a C-terminal domains. (**B**) PrsA and its truncated mutants were expressed in *E. coli* BL21(DE3). Protein A-agarose beads were added and mixed with the cell lysates. Proteins binding to the beads were eluted and detected by immunoblotting using anti-PrsA antibody. (**C**) *E. coli* BL21(DE3)(pET-PrsA) (FL), *E. coli* BL21(DE3)(pET-PrsA-D3) (D3) and *E. coli* BL21(DE3)(pET-PrsA-D4) (D4) were treated with 1% formaldehyde. His-tag proteins were purified from *E. coli* using Ni-NTA beads. Proteins solubilized in sample buffer were incubated at 37 °C for 10 min (lanes 1, 3, 5, 7, 9, 11) or at 95 °C for 30 min (lanes 2, 4, 6, 8, 10, 12) and then separated by SDSȁPAGE, followed with Coomassie blue staining (CBS, lanes 1, 2, 5, 6, 9, 10). PrsA in the protein samples was determined by immunoblotting (IB, lanes 3, 4, 7, 8, 11, 12) using anti-PrsA antibody. Protein bands within boxes were cut and analyzed by LC MS/MS

To delineate the regions in PrsA that interact with SpA, we truncated different regions in His-PrsA and expressed these proteins in *E. coli* BL21(DE3) (Fig. 4A, B upper panel). Protein A-agarose beads were added and mixed with the *E. coli* lysates to determine how the truncations influenced the binding of PrsA to SpA. Immunoblotting analysis revealed that although full-length PrsA (FL) was pulled down by protein A-agarose beads (Fig. 4B, lane 1), PrsA with C-terminal truncations from amino acids 257 (D1) or 147 (D2) was not pulled down by the beads (Fig. 4B, lanes 2, 3). Protein A-agarose beads also did not pull down PrsA with N-terminal 41- (D4), 112- (D5), or 241- (D6) amino acid truncations (Fig. 4B, lanes 5–7). On the other hand, a mutant with a deletion of the PPIase domain from amino acids 147–249 (D3) was pulled down by protein A-agarose beads (Fig. 4B, lane 4). These results indicated that both the N-terminal and C-terminal regions (NC domains) are required for the interaction with SpA.

It has been shown that PrsA of *B. subtilis* and PrsA2 of *L. monocytogenes* form dimers [7, 28, 29]. To elucidate whether PrsA of *S. aureus* can form dimers, formaldehyde cross-linking experiments were performed. First, His-PrsA was expressed in *E. coli* and cross-linked using formaldehyde. Subsequently, the His-tag proteins were purified using Ni-NTA beads and analyzed by SDS-PAGE and immunoblotting. Analysis of SDS-PAGE and Coomassie blue staining (CBS) revealed the monomeric PrsA bands appearing near the 35 kDa position (Fig. 4C, lanes 1, 2). A band near the size 70-kDa was also found on CBS gel (Fig. 4C, lanes 1). The molecular weight of the protein suggested that it may be a PrsA dimer. However, the proteins were not observed after the crosslinking was reversed by heating the proteins at 95 °C (Fig. 4C, lanes 2). Immunoblotting analysis (IB) with an anti-PrsA antibody also obtained a similar result (Fig. 4C, lanes 3, 4). The protein bands near 35 kDa and 70 kDa were cut and analyzed by liquid chromatography-mass spectrometry/mass spectrometry (LC-MS/MS) for protein identification. The results found that the predominant protein in the gel band near 35 kDa is PrsA, accompanied by a minor presence of *E. coli* proteins (Table S2). However, only PrsA was detected in the gel band near 70 kDa, demonstrating the formation of PrsA dimers (Table S2). Furthermore, formaldehyde crosslinking analysis also revealed that the truncated mutant D3, which lacks the PPIase domain and contains only the NC domains of PrsA, can form dimers (Fig. 4C, lanes 5–8, Table S2). Conversely, the truncated mutant D4, which retains the PPIase domain but lacks intact NC domains, did not form dimers (Fig. 4C, lanes 9–12). The results indicate that the NC domains of PrsA are crucial for chaperone activity and dimer formation. Additionally, the bands with sizes other than the monomeric and the dimeric PrsA were observed, which may result from the interaction between PrsA and other proteins.

### Deletion of *PrsA* decreases the binding of immunoglobulins to *S. aureus*

**Fig. 5:**
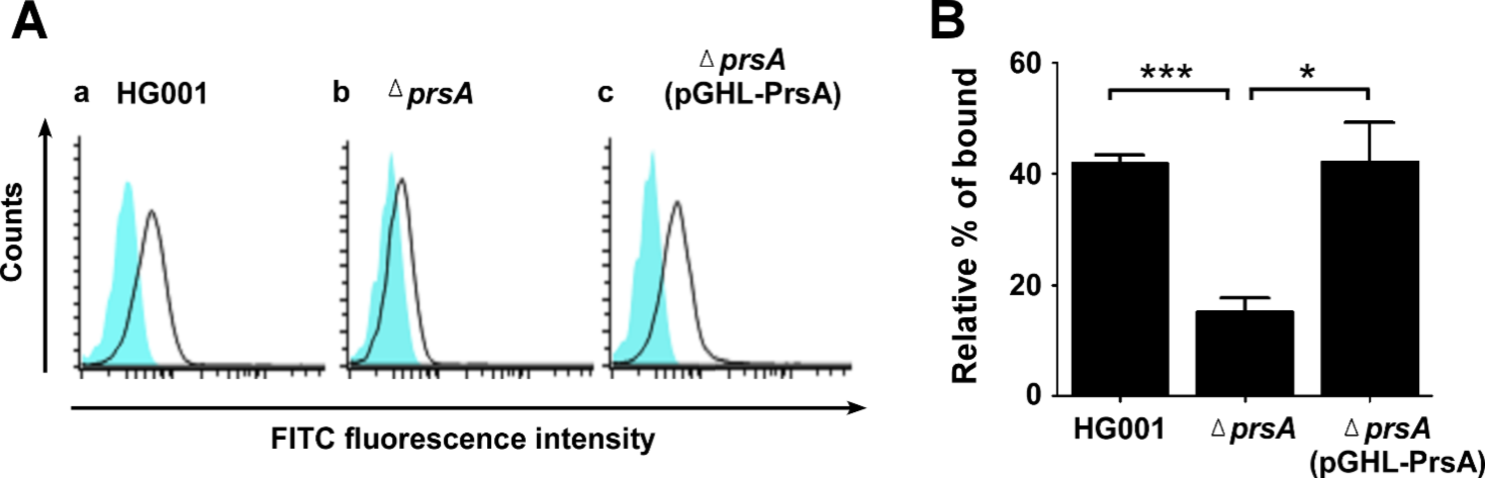
Binding of immunoglobulin IgG to *S. aureus.* (**A**) *S. aureus* HG001, HG001Δ*prsA*, and HG001Δ*prsA*(pGHL-PrsA) were incubated with mouse immunoglobulin IgG and FITC-conjugated anti-mouse IgG antibodies. FITC-labeled *S. aureus* was analyzed by flow cytometry. The white area and the blue area indicate the fluorescence intensity distributions of *S. aureus* strains stained with or without FITC-conjugated antibody. Unstained *S. aureus* (blue area) was used to set the negative population. (**B**) The amount of FITC-labeled *S. aureus* was determined by flow cytometry and expressed as the percentage bound relative to the negative population. Data are presented as the mean of the results from three independent experiments. Error bars denote the standard deviations. Significant differences are denoted *p < 0.05, ***p < 0.001

Protein A is known to bind to the Fc region of immunoglobulins to evade host immunity [23]. To investigate whether PrsA deletion decreased the binding of immunoglobulins to *S. aureus*, flow cytometry was used to quantify the specific Fc-mediated antibody binding to SpA, which is present on the surface of *S. aureus*. *S. aureus* strains were incubated with mouse immunoglobulin IgG and FITC-conjugated anti-mouse IgG antibodies. The binding of IgG to *S. aureus* was analyzed using flow cytometry analysis. The results showed that *S. aureus* HG001 bound significantly more IgG than HG001Δ*prsA*, which had deleted *prsA* and expressed less protein A on its surface (Fig. 5A). The amount of *S. aureus* HG001 binding to IgG was 41.8%, while *prsA* deletion decreased the amount of *S. aureus* HG001 binding to IgG to 15% (Fig. 5B). After *S. aureus* HG001Δ*prsA* was transformed with a PrsA-expressing plasmid, pGHL-PrsA [19], the secretion of protein A was restored, and the amount of *S. aureus* HG001Δ*prsA* (pGHL-PrsA) binding to IgG increased to 42.2% (Fig. 5B).

## Discussion

Gram-positive bacteria utilize the general secretion (Sec) pathway and the twin-arginine translocation (Tat) pathway to transport proteins across the cytoplasmic membrane [30, 31]. The Sec pathway serves as the primary route for exporting most bacterial proteins [32]. The Tat pathway mainly exports folded proteins [30]. In contrast, proteins transported via the Sec pathway are initially in an unfolded state and require proper folding by chaperones or foldases following their translocation across the membrane [2, 31]. The well-known foldases are peptidyl-prolyl isomerases (PPIases), which are a superfamily of molecular chaperons that are ubiquitously expressed in bacteria [33]. PPIases play a vital role in protein folding by catalyzing the *cis/trans* isomerization of peptidyl-prolyl bonds [34]. Numerous studies have revealed the roles of PPIases in modulating the virulence and pathogenicity of various Gram-positive bacterial pathogens [9, 13, 14, 16, 35]. *S. aureus* contains three PPIases, PrsA, PpiB, and trigger factor [35, 36]. Among these three PPIases, only PrsA is membrane-anchored and localized to the space between the cytoplasmic membrane and cell wall [2]. Our earlier proteomic study found that deletion of *prsA* decreases the amount of SpA [19]. This study further investigated how PrsA is involved in the secretion of SpA.

The synthesis of SpA is growth phase-dependent and is expressed during early exponential growth and subsequently suppressed by the global regulatory *agr* system at the late-exponential phase of growth [25]. Our results are in accordance with the expression kinetics of SpA (Fig. 1). Secreted SpA is first anchored to the cell wall and subsequently released into the extracellular medium over time [24, 37]. The study also observed a time-dependent increase in the amount of SpA present in the culture medium of strain HG001. However, in strain HG001Δ*prsA*, throughout the entire growth phase, only a minimal amount of SpA was detected, regardless of whether it was cell wall anchored or released into the medium (Fig. 1). Because PrsA does not affect the expression of SpA at the transcriptional level [19], the results indicated that the absence of PrsA reduced the secretion of SpA during each growth period of the bacteria. Notably, a protein with a molecular weight smaller than SpA was also detected by an anti-SpA antibody in the culture medium. Considering the presence of a second immunoglobulin-binding protein (Sbi) in *S. aureus* [38], it is speculated that the other protein detected in the culture medium is likely Sbi.

Correct protein folding and the avoidance of proteolytic degradation are critical to secreted proteins. Misfolded proteins are unstable and frequently degraded by quality control proteases at the membrane-cell wall interface [2]. Previous studies have indicated that the absence of PrsA in *B. subtilis* results in the accumulation of misfolded proteins between the cell wall and the cell membrane [39], which elicits secretion stress responses and leads to the expression of quality control proteases for degrading misfolded proteins [40, 41]. This study was based on the hypothesis that PrsA is required for the post-translocational folding of SpA and that deficiency of PrsA will result in instability of misfolded SpA and degradation by proteases at the membrane-cell wall interface. Accordingly, in vivo degradation experiments were performed to clarify whether PrsA is responsible for the stability of SpA in *S. aureus*. Previous studies demonstrated that the level of protein A could remain relatively stable over several hours during exponential growth [27]. This phenomenon was verified in this study. Following deletion of *prsA*, the stability of SpA decreased, and the half-life of SpA was shortened (Fig. 2). Based on the results, it was speculated that SpA may rely on PrsA for proper folding into its correct configuration. In the absence of PrsA, unfolded SpA may accumulate in the membrane-cell wall interface and subsequently undergo degradation by proteases. In previous studies, it was shown that the extracellular protease V8 (SspA) and staphopain B (SspB) can cleave SpA [42, 43], suggesting that SspA and SspB may serve as regulatory proteases involved in modulating the secretion of SpA.

In addition to regulating protein secretion through the modulation of protein folding and stability, several studies have suggested that PrsA may also contribute to maintaining the integrity of the cell wall, thereby potentially impacting the process of protein secretion [5, 7, 13, 20]. PBPs are essential factors for preserving the integrity of the bacterial cell wall [44]. It has been reported that PrsA-dependent modulation of PBPs stability is involved in the maintenance of cell wall integrity in *B. subtilis*, *L. monocytogenes* and *S. aureus* [7, 13, 20]. Additionally, in *B. subtilis*, PrsA facilitates protein secretion only under conditions where the cell wall is present [5]. Furthermore, the overexpression of PrsA enhances the secretion of certain extracellular proteins, such as α-amylase, in cells of *Bacillus* and *L. lactis* [45, 46]. These studies proposed that the absence of PrsA may alter the structural components and the charge of the cell wall. While the detailed mechanism of PrsA involved in protein secretion needs to be further investigated, the available information has already demonstrated the significance of PrsA in influencing protein secretion.

As a post-translocational chaperone and PPIase, PrsA may interact with its substrate directly. A pulldown assay in this study provided evidence for the interaction between PrsA and SpA in vivo (Fig. 3). mains. Domain mapping experiments revealed that the binding between PrsA and SpA specifically required the NC domains of PrsA, whereas the PPIase domain was not involved. The results suggest that the NC domains of PrsA might play a role in substrate recognition that is separate from the PPIase activity. The chaperone function of PrsA in *B. subtilis* is known to be independent of its PPIase activity [3, 28]. Interestingly, the PPIase domain of *L. monocytogenes* PrsA2 did not complement the activity of several virulence factors in a *prsA2* deletion mutant, whereas complementation of *prsA2* deletion with the NC domains of PrsA2 restored the hemolytic activity and phospholipase activity [29]. In other Gram-positive bacteria, including *S. mutans*, GAS and *Lactococcus lactis*, the absence of a PPIase motif in their PrsA-like proteins did not affect their chaperone activity [15, 47]. These studies suggest that the PPIase domain and the NC domains of PrsA have distinct functional roles in various bacteria. Furthermore, PrsA of *B. subtilis* has been shown to form a homodimer via its chaperone-like domain, leading to the formation of a bowl-like shaped crevice, which is necessary for its functions [28]. In *L. monocytogenes*, dimerization of PrsA is also required for virulence [48]. Thus far, only limited information exists regarding the structural and functional aspects of *S. aureus* PrsA. We found that PrsA of *S. aureus* also formed dimers (Fig. 4C), implying that the dimerization of PrsA seems to be an essential characteristic closely related to PrsA-associated activity. Previous studies using NMR spectroscopic experiments and crystallographic analysis revealed that *B. subtilis* PrsA forms dimer via its NC domains [28]. This study also found that the NC domains of *S. aureus* PrsA are sufficient to form dimers (Fig. 4C), suggesting their essential roles in both chaperon activity and dimer formation.

Protein A is well known for its capacity to bind immunoglobulins to prevent phagocytosis and evade host immunity [23]. By binding to the Fcγ domains of IgG, SpA protects *S. aureus* against opsonophagocytic clearance and Fc receptor-mediated bacterial killing [49]. The three-dimensional structure of a protein molecule is important to its function. Crystallographic analysis has confirmed that the proper folding of SpA is essential for its interaction with immunoglobulins [50]. This study demonstrated that deletion of *prsA* resulted in reduced immunoglobulins binding to *S. aureus* (Fig. 5), indicating that PrsA plays a crucial role not only in the folding and secretion of SpA but also in the pathogenicity of *S. aureus*.

## Conclusion

*S. aureus* expresses and secretes a large number of virulence factors to cause infection. However, the mechanisms involved in the quality control steps of protein secretion remains unclear. This study demonstrated that PrsA interacts directly with SpA and is involved in modulating its stability and secretion, revealing an important mechanism by which PrsA regulates the virulence of *S. aureus*. Furthermore, PrsA is a surface protein and has higher antigenic properties [51], which means it is accessible to the immune system. In summary, as a surface-exposed antigen and a virulence factor regulator, PrsA is a promising candidate for developing vaccines against *S. aureus*.

## Materials and methods

### Bacterial strains and culture conditions

The bacterial strains and plasmids used in this study are listed in Table 1. Bacterial strains were cultured in tryptic soy broth (TSB) or agar (TSA) (Oxoid, Basingstoke, United Kingdom). Antibiotic-resistant colonies were selected on media that contained spectinomycin (Spec, 100 µg/ml), erythromycin (Em, 5 µg/ml), chloramphenicol (Cm, 10 µg/ml), tetracycline (Tc, 5 µg/ml) or ampicillin (Ap, 100 µg/ml).

**Table 1:** Bacterial strains and plasmids used in this study

Strains or Plasmids	Description	Reference
Strain		
*E. coli*		
EPI300	a host for cloning	Epicentre
BL21(DE3)	a host for expressing recombinant proteins	[51]
*S. aureus*		
RN4220	a restriction-defective strain derived from *S. aureus* NCTC8325-4 was used as a host for cloning	[52]
HG001	a derivative of *S. aureus* NCTC8325	[53]
HG001Δ*prsA*	an isogenic mutant derived from strain HG001, containing a deletion in *prsA*; *prsA*::*spec*	[19]
HG001Δ*prsA* (pGHL-PrsA)	HG001Δ*prsA* complemented with pGHL-PrsA; restored for PrsA production	[19]
SA113Δ*spa*	an isogenic mutant derived from strain SA113, containing a deletion in *spa*; *spa*::*erm*	[53]
**Plasmid**		
pGHL7	a shuttle vector carrying a pC194 *ori*, a ColE1 *ori*, an Ap resistance gene, and a Cm resistance gene, was used for cloning	[19]
pGHL-PrsA	a derivative of pGHL7, containing *prsA*	[19]
pGHL-SpA-His	a derivative of pGHL7, containing a DNA fragment that encodes the full-length protein A with a C-terminal His-tag	This study
pET-PrsA	a derivative of pET-30b, containing a DNA fragment that encodes the full-length PrsA (amino acid 1 to 320)	[19]
pET-PrsA-D1	a derivative of pET-PrsA, containing a DNA fragment that encodes amino acids 1 to 256 in PrsA	This study
pET-PrsA-D2	a derivative of pET-PrsA, containing a DNA fragment, which encodes amino acids 1 to 146 in PrsA	This study
pET-PrsA-D3	a derivative of pET-PrsA, containing a DNA fragment, which encodes amino acids 1 to 146 and 251 to 320 in PrsA	This study
pET-PrsA-D4	a derivative of pET-PrsA, containing a DNA fragment, which encodes amino acids 42 to 320 in PrsA	This study
pET-PrsA-D5	a derivative of pET-PrsA, containing a DNA fragment, which encodes amino acids 114 to 320 in PrsA	This study
pET-PrsA-D6	a derivative of pET-PrsA, containing a DNA fragment, which encodes amino acids 243 to 320 in PrsA	This study

### Preparation of cell wall-associated proteins and exoproteins

Bacterial cell wall-associated proteins and exoproteins were isolated as described previously [19]. Briefly, overnight cultures of all strains were inoculated in fresh TSB to an OD_578_ of 0.05 and subcultured at 37 °C with shaking for several hours. The bacterial culture at an OD_578_ of 5.0 (approximately 5 × 10^8^ CFU) was collected. The supernatant was removed for the extraction of exoproteins. The remaining cell pellets were divided into two portions, one part used for the extraction of cell wall-associated proteins and the other part used for preparing cell lysates. The proteins in the culture supernatant were concentrated using Amicon Ultra centrifugal filters (Millipore, Billerica, MA, United States). Cell wall-associated proteins were extracted from the cell pellets that were washed twice with digestion buffer (50 mM Tris-HCl, pH 7.6, 20 mM MgCl_2_) and treated with digestion buffer containing 35% sucrose, protease inhibitor cocktail (Sigma‒Aldrich), and 0.4 mg lysostaphin (Sigma Aldrich, St. Louis, MO, USA) for 30 min at 37 °C. After centrifugation at 2500 × *g* for 15 min at 4 °C, the supernatant was treated with 20 µg/ml DNase I and RNase A for 30 min at room temperature and was then centrifuged at 18,000 × *g* for 30 min at 4 °C. The cell wall-associated proteins in the supernatant were then concentrated using Amicon Ultra centrifugal filters. The concentrated proteins were adjusted to the same volume, and equal volumes of concentrated proteins were used for immunoblotting analysis. The cell lysates were used as the sample control.

### Plasmid construction

The primers used in this study are listed in Table S1. To construct pGH-SpA-His, *S. aureus* HG001 chromosomal DNA was used as the template to amplify the DNA fragment containing full-length *spa* by PCR using the primer set His-Spa-F/His-Spa-R. The DNA fragment was cut by EcoRI and XbaI and then inserted into the EcoRI-XbaI sites of pGHL7 [19]. To generate the deletions of *prsA*, the plasmid pET-PrsA [19] that contains full-length *prsA* was subjected to inverse PCR using the primer sets D1-F/D1-R, D1-F/D2-R, D3-F/D3-R, D4-F/D4-R, D5-F/D4-R and D6-F/D6-R (Table S1) to generate pET-PrsA-D1, pET-PrsA-D2, pET-PrsA-D3, pET-PrsA-D4, pET-PrsA-D5, and pET-PrsA-D6 (Table 1).

### Expression and purification of his-tagged PrsA

The His-tagged proteins were expressed and extracted from *E. coli* BL21(DE3) according to a method described previously [19]. In brief, *E. coli* BL21(DE3) containing plasmids that expressed full-length PrsA or its truncated mutants were cultured in TSB medium to mid-log phase. The expression of His-tagged proteins was induced with 0.1 mM isopropyl *β*-d-1 thiogalactopyranoside (IPTG) and then the strains were incubated for an appropriate time. After induction, the cells were harvested by centrifugation and suspended in a lysis buffer that contained 50 mM NaH_2_PO_4_ (pH 8.0), 300 mM NaCl, 10 mM imidazole, and a protease inhibitor cocktail (Sigma‒Aldrich). The cells were homogenized with glass beads using a homogenizer (Analytik Jena AG, Uberlingen, Germany). The lysate was centrifuged at 16,000 × *g* at 4 °C for 30 min. The His-tagged proteins in the lysate were purified using Ni-NTA beads (Qiagen, Valencia, CA, USA) according to the manufacturer’s instructions.

### Detecting the stability of SpA in cells

An in vivo protein degradation assay was performed according to a method described elsewhere [52] with some modifications. Briefly, overnight cultures of all strains were inoculated into 100 ml fresh TSB to an OD_578_ of 0.05 and subcultured at 37 °C with shaking for 5 h. The bacterial culture was supplemented with erythromycin at a final concentration of 100 µg/ml to stop protein translation. Cells were continuously incubated in a 37 °C water bath, and the culture was taken at 30 min intervals for 240 min. The cell wall-associated proteins were extracted as described above. The amount of SpA was detected by immunoblotting using an anti-protein A monoclonal antibody (Sigma-Aldrich). Protein bands were quantified using ImageJ software, and relative intensities were plotted. The half-life of SpA was calculated from the exponential regression analysis.

### Immunoblotting

Proteins were separated by sodium dodecyl sulfate‒polyacrylamide gel electrophoresis (SDS‒PAGE) and electrotransferred onto PVDF membranes (Merck, Taipei, Taiwan) at 90 V for 1 h. The membranes were stained using 0.2% Ponceau S (dissolved in 1% acetic acid) to enable proteins visualization on the membranes, thereby confirming the successful transfer and equal loading of proteins. After washing with distilled water, membranes were probed with the appropriate antibodies and reacted with chemiluminescent reagents (ECL, Thermo Fisher Scientific). The signals of protein bands on PVDF membranes were visualized by exposure to X-ray film (Kodak Co., Rochester, NY, USA) or by using an ImageQuant LAS 4000 system (GE Healthcare Bio-Sciences, Pittsburgh, PA, USA).

### Protein‒protein interaction assay

The full-length His-tagged PrsA and its deletion derivatives were purified from *E. coli* and added to 35 µl of protein A agarose bead suspension (Roche). The mixture was incubated at 4 °C for 2 h on a rotator. After centrifugation, the agarose beads were washed three times in 500 µl of wash buffer (50 mM Tris-HCl, pH 7.6, 150 mM NaCl, 5 mM EDTA, pH 8.0, 0.5% Nonidet P-40) and resuspended in 20 µl of sample buffer (200 mM DTT, 4% SDS, 100 mM Tris, 20% glycerol, 0.2% bromophenol blue). Proteins were extracted from the beads by heating at 95 °C for 5 min and analyzed using immunoblotting.

### Cross-linking of PrsA

Protein cross-linking was performed with formaldehyde as described by Jensen et al. [53] with minor modifications. Briefly, bacteria were cultured to the exponential phase and pelleted by centrifugation. The cell pellets were washed once with 1 ml 0.1 M sodium phosphate buffer (pH 6.8) and resuspended in the same buffer containing 1% formaldehyde for 5 min at room temperature. Cells were then pelleted, washed twice in phosphate buffer. The His-tagged proteins were extracted from *E. coli* BL21(DE3) and purified using Ni-NTA beads, following the methods as described above. The proteins in sample buffer were heated either at 37 °C for 10 min or at 95 °C for 30 min to break the formaldehyde cross-links and subsequently analyzed by SDS‒PAGE and immunoblotting using an anti-PrsA antibody.

### Flow cytometry analysis

Overnight cultures of all strains were inoculated in fresh TSB to an OD_578_ of 0.05. After culturing at 37 °C with shaking for 6 h, the cells were centrifuged, washed, and resuspended in 2% BSA-PBS. Approximately 10^5^ CFU of bacteria were incubated with mouse immunoglobulin IgG at 37 °C for 30 min. Bacteria were then washed and incubated with FITC-conjugated anti-mouse IgG at 4 °C for 30 min. The binding of immunoglobulins to *S. aureus* was detected by a Guava EasyCyte flow cytometer (Merck Millipore, Germany). Data were analyzed using Guava® InCyte™ software.

### Statistical analysis

Data were analyzed by two-tailed Student’s *t*-test using GraphPad Prism software version 8.0 (GraphPad Software, San Diego, CA, USA). A p-value of < 0.05 was considered statistically significant. Data are presented as the mean ± SD.

### Electronic supplementary material

Below is the link to the electronic supplementary material.

Supplementary Material 1

## Data Availability

The authors confirm that the data supporting the findings of this study are available within the article.
