# Supplementary material for: Staphylococcus aureus foldase PrsA contributes to the folding and secretion of protein A
Source: BMC Microbiol. 2024 Apr 2;24:108. doi: 10.1186/s12866-024-03268-7 (PMC10986000; doi:10.1186/s12866-024-03268-7)
Supplement: Supplementary file 1 — Supplementary Material 1 [file 12866_2024_3268_MOESM1_ESM.pdf]

Supplementary Materials

Table S1 Primers used in this study

Primer	Sequence 5'-3'
His-Spa-F	AGGCATGAATTCATGGATGACGCGCAGCAAGACCATGCTGAACAATTAT
His-Spa-R	TGTTCTAGATTAGTGGTGGTGGTGGTGGTGTAGTTCGCGACGACGTCCA
D1-F	GCGGACAATCCGGCATGAGCCAAGAATTC
D1-R	CGGGAATTCTTCAGGCTTTGTTTTTCACTG
D2-R	GCCGAATTCATGTGTGAAGCTTTCTTGCTG
D3-F	CCCGGATCCAGTGAAAA ACAAAGCCTGAA
D3-R	CCCGGATCCAATGTGTGAAGCTTTCTTGCT
D4-F	GGGGGTACCACAGTTGCAGATACAATGAAA
D4-R	AAAGGTACCCAGATCTGGGCTGTCCATGTG
D5-F	GGGGGTACCGAAAATTTACGTACTGCTGCT
D6-F	AACGGATCCGCTGATAAACCAACAGACTT
D6-R	AAAGGATCCGGTACCCAGATCTGGGCTGT

Table S2. Proteins identified in protein bands 1-3 of Figure 4C.

Protein band	Accession No.	Protein name	Coverage [%]	[a]No. of unique peptides	MW [kDa]	Score Sequest HT
**1**	Q2G2S6	Foldase protein PrsA	74	42	35.6	1017
	P0AEW9	1-phosphofructokinase (*Escherichia coli*)	6	2	33.7	4.5
**2**	Q2G2S6	Foldase protein PrsA	53	24	35.6	317.7
**3**	Q2G2S6	Foldase protein PrsA	32	12	35.6	109

a: the protein identification with at least two unique peptides

Supplemental Fig. S1

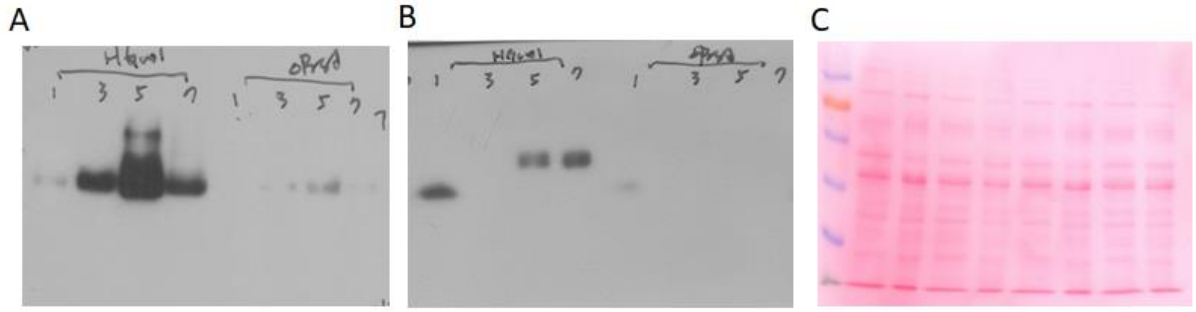

Supplemental Fig. S1. The full-length blots and loading controls of Figure 1. The full-length blots of Figure 1A (A) and Figure 1B (B). The PVDF membranes were stained with 0.2% Ponceau S solution as loading controls of Figure 1 (C).

Supplemental Fig. S2

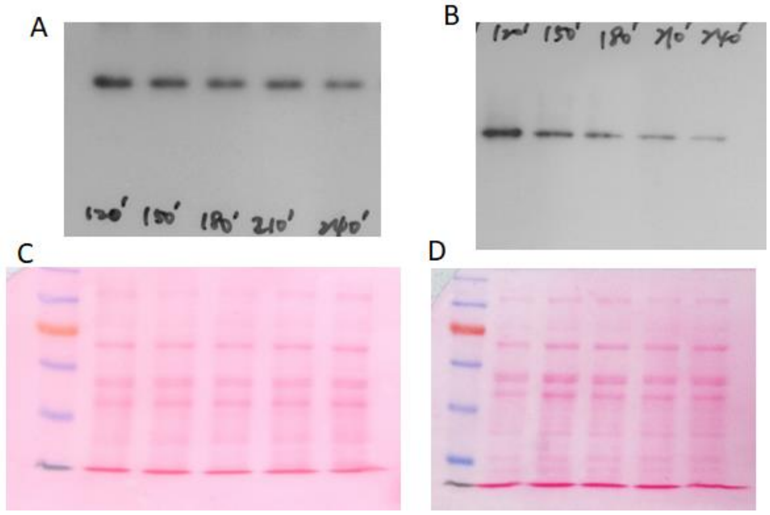

Supplemental Fig. S2. The full-length blots and loading controls of Figure 2. The full-length blots of Figure 2A (A) and Figure 2B (B). The PVDF membranes were stained with 0.2% Ponceau S solution as loading controls of Figure 2A (C) and Figure 2B (D).

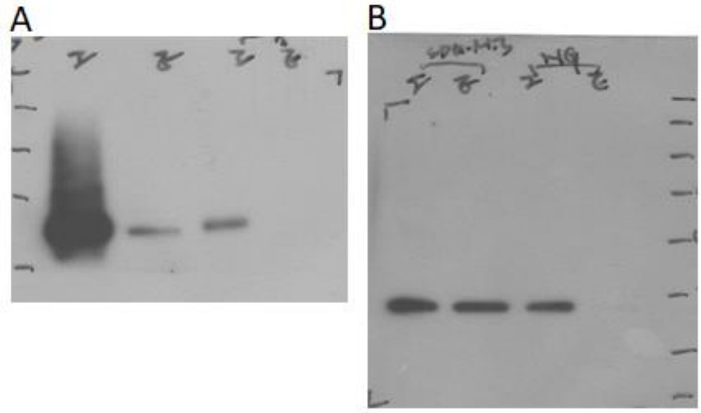

Supplemental Fig. S3. The full-length blots and loading controls of Figure 3. The full-length blots of Figure 3 upper panel (A) and lower panel (B).

Supplementary Methods

Proteomic analysis by LC-MS/MS

The mass spectrometry-based proteomics analysis was performed by the Clinical Proteomics Core Laboratory of Chang Gung Memorial Hospital at Linkou, Taiwan.

Protein In-gel digestion

The protein bands were cut into small pieces (~1 mm^3) and reduced and alkylated with DL-Dithiothreitol (Sigma-Aldrich, USA) and iodoacetamide (Sigma-Aldrich, USA) reagents, respectively, and then digested with sequencing grade trypsin (Promega, USA). Following the in-gel digestion procedures, the peptides were desalted by Source 15rpc (GE Healthcare,

SWEDEN).

Tandem mass spectrometry-based protein identification

Tandem mass spectrometry analysis was performed on Q Exactive^{TM} HF mass spectrometer (Thermo Fisher, San Jose) coupled with a Thermo Scientific™ UltiMate™ 3000 RSLCnano HPLC System. The peptide mixtures are directly loaded onto a 50-cm analytic column (EASY-Spray™ C18 Column), and separated by a gradient with gradually increased buffer B (80 % acetonitrile in 0.1 % formic acid) at a flow rate of 200 nL/min over about 90 min. The peptide spectra were acquired in positive ion mode with a data-dependent acquisition. The top abundant fifteen precursor ions within 400-1600 m/z scan range were dynamically selected for further fragmented in high collision dissociation (HCD) mode with normalized collision energy set to 28. In Full MS scan, the resolution was set to 60000 at m/z 200, AGC target to 3e6, maximum inject time to 30ms. In MS/MS scan, the resolution was set to 15000, AGC target to 1e5, maximum inject time to 100ms. The release of dynamic exclusion of the selected precursor ions was set to 20 sec.

Analysis of MS-generated proteomic data by Proteome Discoverer software and SEQUEST search algorithm

The MS raw files are uploaded into Proteome Discoverer (version 2.4, Thermo Fisher Scientific, MA, USA) to generate a peak list for the following protein identification analysis by using *SEQUEST search* algorithm against *E. coli* (strain K12) and S*taphylococcus aureus* (strain NCTC 8325 / PS 47) protein sequences of Uni-PROT database (released in Jan, 2024). For protein identification, Carbamidomethyl at Cys was used as the fixed modification, oxidation at Met, Gln to pyro-Glu at peptide N-terminus, acetylation at protein N-terminus set as dynamic modifications, maximum missing cleavage sites with 2, 10 ppm for MS tolerance and 0.03 Da for MS/MS tolerance were allowed for database searching. The peptide and protein identifications with false discovery rate less than 1% are accepted. To improve confidence, the protein identification with at least three unique peptides.
